# A simplified nasopharyngeal swab collection procedure for minimizing patient discomfort while retaining sample quality

**DOI:** 10.3389/fpubh.2023.1066934

**Published:** 2023-03-23

**Authors:** Tina Uršič, Rok Kogoj, Jaka Šikonja, Monika Jevšnik Virant, Miroslav Petrovec

**Affiliations:** Institute of Microbiology and Immunology, Faculty of Medicine, University of Ljubljana, Ljubljana, Slovenia

**Keywords:** nasopharyngeal swab, collection procedure, sample quality, discomfort score, Ubiquitin C

## Abstract

A nasopharyngeal swab (NPS) is the most frequently collected sample type when molecular diagnosis of respiratory viruses, including SARS CoV-2, is required. An optimal collection technique would provide sufficient sample quality for the diagnostic process and would minimize the discomfort felt by the patient. This study compares a simplified NPS collection procedure with only one rotation of the swab to a more standard procedure with five rotations. Swabs were collected from 76 healthy volunteers by the same healthcare professional on 2 consecutive days at a similar hour to minimize variability. The number of Ubiquitin C copy number per sample was measured by real-time quantitative PCR and patient discomfort was assessed by questionnaire. No statistically significant difference (*p* = 0.15) was observed in the Ubiquitin C copy number per sample between a NPS collected with one rotation (5.2 ± 0.6 log *UBC* number copies/sample) or five rotations (5.3 ± 0.5 log *UBC* number copies/sample). However, a statistically significant difference was observed in discomfort between these two procedures, the second being much more uncomfortable. Additional analysis of the results showed a weak correlation between discomfort and the number of human cells recovered (Spearman's rho = 0.202) and greater discomfort in younger people. The results of this study show that a NPS collected with one slow rotation has the same quality as a NPS collected with five rotations. However, the collection time is shorter and, most importantly, less unpleasant for patients.

## 1. Introduction

A nasopharyngeal swab (NPS) is the most commonly collected specimen when laboratory diagnosis of respiratory viral infections, including COVID-19, is required. As an appropriate NPS collection technique is critical for reliable results and for minimizing patient discomfort during the procedure (1), so is the proper use of personal protective equipment to minimize the risk of health care professionals' infection (2). Current recommendations for obtaining NPS are not uniform. The World Health Organization (WHO) recommends leaving the swab in place for a few seconds after reaching the nasopharynx before withdrawing it (3), the Center for Disease Control and Prevention (CDC) recommends “gently rubbing and rolling” the swab after reaching the nasopharynx and leaving it in place for several seconds before withdrawing it (4). To further address these issues and provide additional guidance, Marty et al. published an article with practical instructions and a descriptive video in *The New England Journal of Medicine* early in the pandemic (5). Nevertheless, we wondered if the procedure could be further changed to reduce the proportion of patients that find it frustrating or even painful, and simplified without compromising the quality of the sample.

The aim of this study was to investigate whether the NPS collection could be simplified to only one rotation with no waiting period in the nasopharynx yet retain the sample quality but decrease the discomfort felt by the participants.

## 2. Methods

### 2.1. Study participants, sample collection, and processing

In this study, we collected 3 NPS samples from 76 healthy adults without any respiratory symptoms over the course of 7 days. A single healthcare provider collected all NPSs to minimize differences in the swab collection procedure. We used the CITOSWAB VTM (Nal Von Minden, Regensburg, Germany) collection kit containing 3 ml of viral transport medium. NPS were collected on 2 consecutive days at around the same time. On the 1st day, sample A was collected with one rotation and sample B with five rotations one after another with enough time in between to allow the participant to recover from the discomfort experienced. On the 2nd day, only a NPS with five rotations (sample C) was collected. The detailed method for sample A collection was:

- Gentle insertion of the swab through the nostril;
- Simultaneous pushing and rotating of the swab 5–7 cm to the nasopharynx and immediate removal with simultaneous rotation during withdrawal amounting to one full rotation;

Samples B and C were collected by following previously published recommendations (5).

Study participants were asked to rate their discomfort immediately after collection on a scale from 1 representing absence or no discomfort, linearly to 10 representing unbearable discomfort.

Samples were blinded and processed within 3 h after sample collection. Total nucleic acids (NA) were extracted using a MagNA Pure Compact instrument (Roche, Mannheim, Germany). The swab collection quality was assessed by quantifying the amount of human UBC gene copies per sample (6) by using LightMix^®^ Kit SARS-CoV-2 E+N UBC (TibMolbiol, Berlin, Germany) with an in-house UBC quantification standard. The result was finally expressed as log [UBC copies/sample] taking into account volume calculations, 200 μl of sample volume and 100 μl of elution volume.

### 2.2. Statistical methods

Differences in sample quality expressed as log [UBC copies/sample] were evaluated with parametric tests. Discomfort scores, classified as ordinal data, were compared with non-parametric tests. Therefore, for in-group and between-group comparisons, a paired *t*-test (or Wilcoxon signed-rank test) and independent samples *t*-test (Mann–Whitney *U*-test) were used, respectively. The strength of the association between discomfort scores and log [UBC copies/sample] was assessed using Spearman's rank-order correlation. A value of *p* ≤ 0.05 was considered statistically significant in all tests. Continuous data, such as log [UBC copies/sample], are presented as mean ± standard deviation, whereas ordinal data (discomfort score) are presented as median (first–third quartile).

## 3. Results

**Figure 1:**
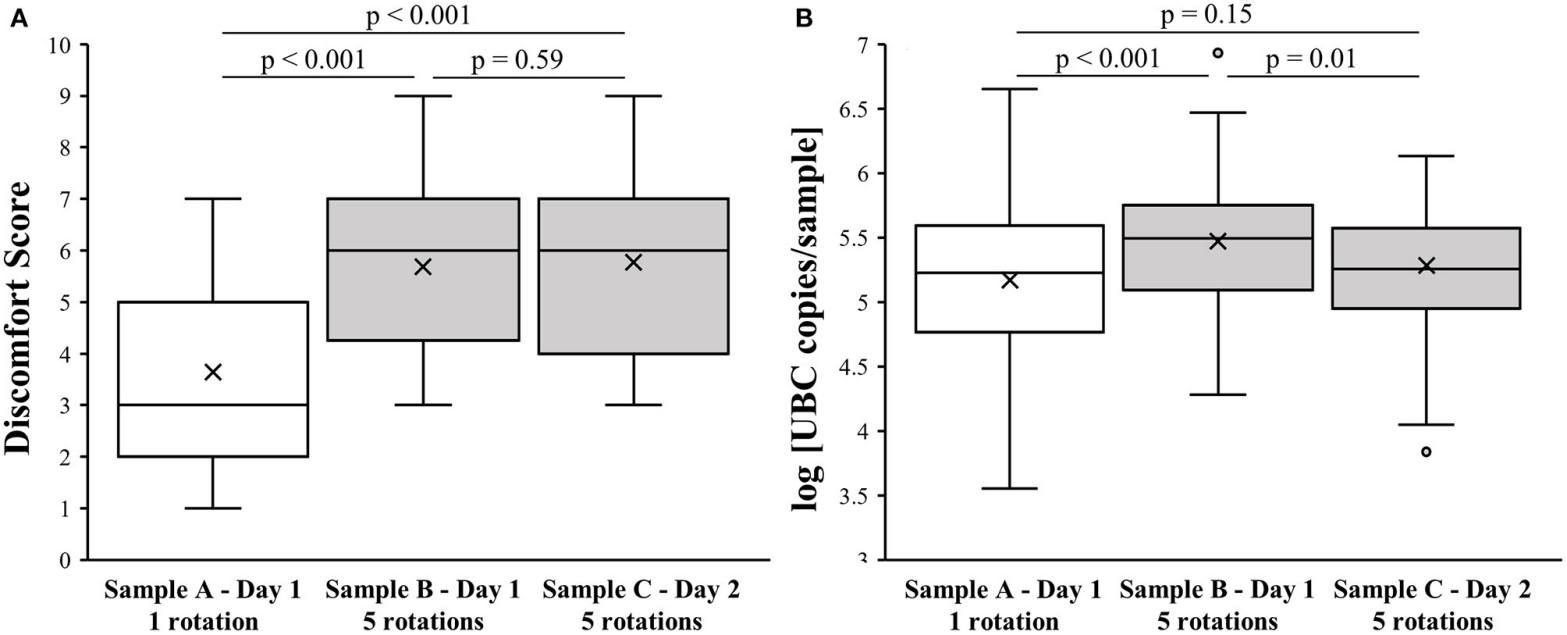
Effect of NPS collection method on level of discomfort and sample quality. **(A)** Association of NPS collection technique with discomfort score. **(B)** Association of NPS collection technique with log [UBC copies/sample].

The mean age of the 76 study participants was 36 ± 11 years, and 56 (74%) were female. The observed median discomfort score (DS) for sample A was 3 (first-third quartile; 2–5), whereas for sample B it was 6 (first-third quartile; 4.25–7) and for sample C it was also 6 (first-third quartile; 4–7), as shown in Figure 1A. The DS of sample A was significantly lower when compared to samples B and C (*p* < 0.001). No statistically significant difference was observed between samples B and C (*p* = 0.59).

There was no significant difference in the log [UBC copies/sample] between samples A and C (5.2 ± 0.6 vs. 5.3 ± 0.5; *p* = 0.15). However, sample B had higher log [UBC copies/sample] values (5.5 ± 0.5) compared to samples A and C (*p* < 0.001 and *p* = 0.01, respectively), as presented in Figure 1B.

Although younger participants ( ≤ 35 years) reported significantly higher DS in comparison to older participants (sample A, median: 4 vs. 3, *p* = 0.007; sample B, median: 6 vs. 5, *p* = 0.02; sample C, median: 7 vs. 5.5, *p* = 0.056), the two age groups did not differ in mean log [UBC copies/sample]. Moreover, comparable DS were observed between men and women (sample A, median: 3.5 vs. 3, *p* = 0.843; sample B, median: 5.5 vs. 6, *p* = 0.815; sample C, median: 6 vs. 6, *p* = 0.728) as were also log [UBC copies/sample] *p* > 0.1.

**Figure 2:**
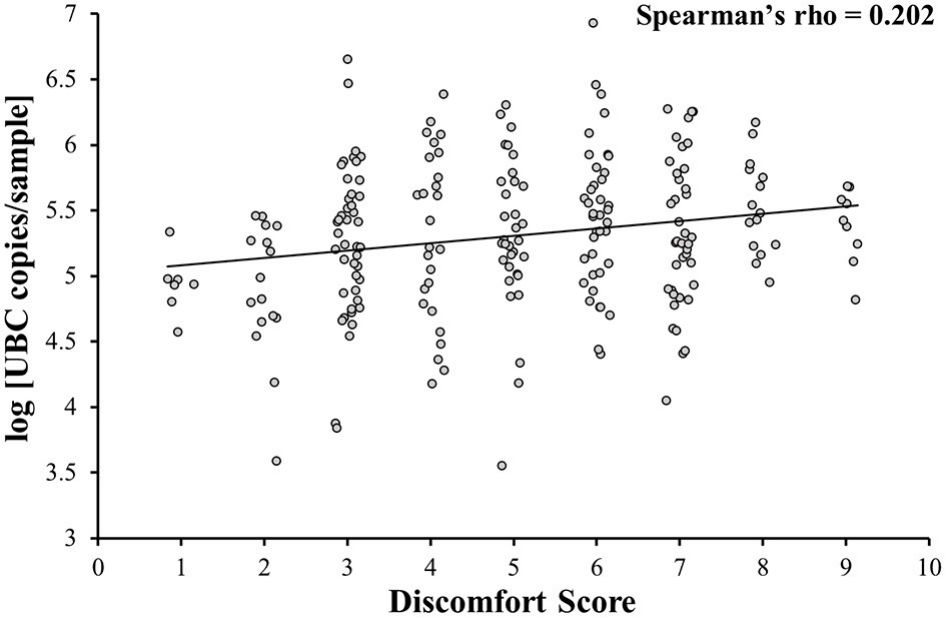
Correlation between the level of discomfort and sample quality. Discomfort is weakly correlated with sample quality (samples A, B, and C together).

A weak positive correlation between DS and log [UBC copies/sample] was observed (Spearman's rho = 0.202; *p* = 0.002; Figure 2). Additional analysis on samples A, B and C together showed that NPS collected with minimal subject discomfort (DS < first quartile) had a lower log [UBC copies/sample] than NPS collected with high subject discomfort (DS > third quartile; 4.9 ± 0.4 vs. 5.5 ± 0.3; *p* < 0.001). When looking separately, only sample A showed that NPS collected with minimal subject discomfort (DS < first quartile) had a lower log [UBC copies/sample] than NPS collected with high subject discomfort (4.9 ± 0.2 vs. 5.7 ± 0.4; *p* < 0.001) and no link between DS and log [UBC copies/sample] was observed in samples B (5.4 ± 0.6 vs. 5.3 ± 0.4; *p* = 0.42) and C (5.2 ± 0.5 vs. 5.4 ± 0.3; *p* = 0.22).

## 4. Discussion

The results of this study show no statistically significant difference in the UBC gene copies/sample between NPS collected with one or five rotations (sample A vs. sample C). These results are also supported by the study from Kinloch et al. (7). They similarly found no difference in DNA/RNA recovery between an “in–out” NPS and a NPS with 10 s of rotation. Our study found a significantly higher DS of NPS with five rotations compared to one rotation NPS. Interestingly, in contrast to our results, Kinloch et al. found no differences in discomfort between the procedures they used (7).

Despite all efforts for uniformity of the collection procedure, differences in log [UBC copies/sample] values were still found between study participants, which may indicate the diversity of the nasal mucosa and anatomy of the individuals (7, 8). In the study by Kinloch et al., RPP30 and RNase *p*-values also varied markedly regardless of the swab collection technique (7).

Although only a weak correlation between DS and log [UBC copies/sample] was observed between the participants that reported almost no discomfort and those that reported the highest DS, the difference between log [UBC copies/sample] reached a statistically significant value. This observation theoretically means that samples with higher human cell counts would have an impact on specific target (pathogen) detection sensitivity, however due to the study design such a conclusion can't be drawn. Similar results were also observed by Kinloch et al., who showed statistically significant higher DNA/RNA recovery together with higher DS in Asian participants of their study population (7, 8).

Finally, sample B was initially collected because we wanted to gain insight into the DS and quality of the sample if one sample is collected right after another. Contrary to expectations, we found UBC copies/sample in sample B higher than in sample A and even sample C, however, the difference was rather small. We believe that minor trauma to the nasal pseudostratified ciliated epithelium may have occurred with the collection of sample A, which could explain the higher UBC copies/sample in sample B.

A few limitations of the study must be kept in mind when interpreting the results. Firstly, the study population was rather small, due to difficulties of acquiring willing participants after 2 years of frequent NPs collections for SARS-CoV-2 screening. Secondly, only adults were included in the study, therefore the results do not necessarily apply to children, especially young children who do not yet understand why NPs is being collected and are usually already under stress at the collection site which makes them hindered to give an rational DS score. Thirdly, the study was performed on healthy individuals and different results might occur in individuals with acute respiratory infection, who usually secrete increased amounts of mucus and cellular material. Finally, if NPSs were collected by multiple healthcare professionals, although with the same protocol, additional variance might have been observed in both, quality and DS. Future studies should address these questions.

## 5. Conclusion

In conclusion, this study shows that a simplified NPS collection procedure has the same recovery of human DNA as a NPS collected with five rotations. However, the collection time is shorter and the experience is less unpleasant for patients. We firmly believe that the NPS collection procedure could be improved globally (3, 4, 7).

## Data availability statement

The raw data supporting the conclusions of this article will be made available by the authors, without undue reservation.

## Ethics statement

The studies involving human participants were reviewed and approved by National Medical Ethics Committee of Slovenia (No. 0120-281/2022/3). The patients/participants provided their written informed consent to participate in this study.

## Author contributions

TU: conceptualization, methodology, visualization, and writing—original draft preparation. RK: writing—reviewing and editing, supervision, and reviewing. JŠ: software, data curation, and writing—original draft preparation. MJ: investigation, validation, and reviewing. MP: supervision, reviewing, and editing. All authors contributed to the article and approved the submitted version.
